# Insight Into the Prospects for RNAi Therapy of Cancer

**DOI:** 10.3389/fphar.2021.644718

**Published:** 2021-03-16

**Authors:** Zhili Tian, Guohui Liang, Kunli Cui, Yayu Liang, Qun Wang, Shuangyu Lv, Xiaoxia Cheng, Lei Zhang

**Affiliations:** ^1^ Institute of Molecular Medicine, Henan University, Kaifeng, China; ^2^ School of Clinical Medical Sciences, Henan University, Kaifeng, China; ^3^ School of Basic Medical Sciences, Henan University, Kaifeng, China; ^4^ School of Stomatology, Henan University, Kaifeng, China

**Keywords:** RNAi, cancer, delivery systems, nanoparticles, exosomes

## Abstract

RNA interference (RNAi), also known as gene silencing, is a biological process that prevents gene expression in certain diseases such as cancer. It can be used to improve the accuracy, efficiency, and stability of treatments, particularly genetic therapies. However, challenges such as delivery of oligonucleotide drug to less accessible parts of the body and the high incidence of toxic side effects are encountered. It is therefore imperative to improve their delivery to target sites and reduce their harmful effects on noncancerous cells to harness their full potential. In this study, the role of RNAi in the treatment of COVID-19, the novel coronavirus disease plaguing many countries, has been discussed. This review aims to ascertain the mechanism and application of RNAi and explore the current challenges of RNAi therapy by identifying some of the cancer delivery systems and providing drug information for their improvement. It is worth mentioning that delivery systems such as lipid-based delivery systems and exosomes have revolutionized RNAi therapy by reducing their immunogenicity and improving their cellular affinity. A deeper understanding of the mechanism and challenges associated with RNAi in cancer therapy can provide new insights into RNAi drug development.

## Introduction

**FIGURE 1:**
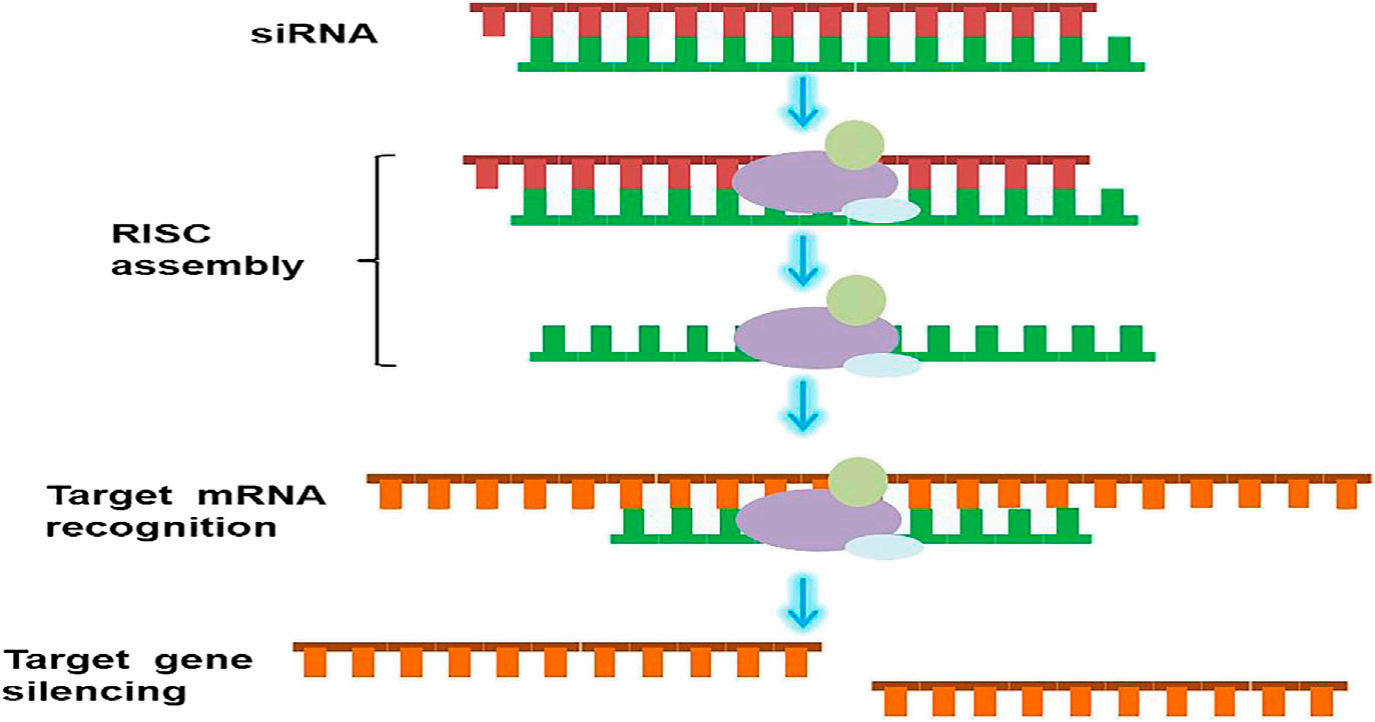
Schematic illustration of RNAi mechanism. siRNA binds to RISC after entering cytoplasm. The antisense chain is loaded onto the RISC, and the sense chains are discarded. The antisense chain then causes the target mRNA to be specifically cut and degraded.

Cancer is one of the most fatal diseases with poor prognosis and scarce solutions. Cancers typically develop from the epithelium and are commonly seen in every part of the body. The current cancer statistics (2020) show that the mortality of cancer has been decreasing since 1990, however, in larger populations, there are still many patients with no access to more efficient and less painful treatment (Siegel et al., 2020). Traditional surgery, radiotherapy, and chemotherapy have been the approaches used for cancer treatment. Nevertheless, these approaches are associated with long-term financial, physical, and mental burden. Thus, new strategies are urgently needed to improve treatment outcomes and reduce these burdens. The discovery of RNAi genetic therapies has led to the treatment of several incurable diseases (Romano and Macino, 1992). RNAi treatments elicit their pharmacological effect *via* non-coding RNAs (ncRNAs), which regulate events of the cell instead of translating into proteins, and with small interfering RNAs (siRNAs) and microRNAs (miRNAs), RNAi acts rapidly. siRNA is a double-stranded ncRNA with a length of 20–25 base pairs that are loaded onto the RNA-induced silencing complex (RISC), to degrade and cleave the mRNA before translation (Watts and Corey, 2012; Ferguson et al., 2020). siRNAs degrade mRNAs, which contain complementary nucleotide sequences, after transcription to interfere with the expression of specific genes (Figure 1). This is a key step in the development of most RNAi therapies. On one hand, with precision and accuracy, siRNAs are regarded as an accessible option for many genetic or orphan diseases. On the other hand, given their easy degradability, the focus of RNAi therapies is ensuring the therapeutic concentrations of the drug reach the target sites on time. The double-stranded miRNA was then loaded into the RISC. Afterward one single-stranded miRNA was degraded, and the other mature single-stranded miRNA molecule was paired with the target mRNA sequence to regulate gene expression (Watts and Corey, 2012; Bobbin and Rossi, 2016).

Owing to the feature of regulating gene expression, RNAi has been deemed to be the ideal strategy for cancer treatment apart from conventional long-period strategies. RNAi adjusts cancerous cells from the inside of the cell, which improves the efficiency of the cancer treatment. and have boosted the resurgence of RNAi technology (Bobbin and Rossi, 2016). In the 2016 World Health Organization’s classification of tumors of the central nervous system, glioblastoma (GBM) was categorised as grade IV astrocytoma, which is a commonly seen lethal brain tumor, and many aptamers have already been tested as therapeutic agents in GBM (Watts and Corey, 2012; Louis et al., 2016; Nuzzo et al., 2020).

Researchers hypothesis that miRNAs are significant regulators in breast cancer as well (Watts and Corey, 2012; Abdalla et al., 2020). Renoir JM has revealed the relationship between breast cancer and miRNAs, leading to the discovery of potential treatments. This review discusses the use of oligonucleotides in cancer therapy in detail. The current oligonucleotide drugs are not well tolerated because of their toxicity to noncancerous cells. Due to challenges such as off-target effects, the therapy has a short duration, limiting treatment efficiency and satisfaction. For this therapy to achieve its therapeutic objective, these off-target effects must be addressed. Many carriers have been designed to address these problems. For example, lipid-based delivery systems provide high efficiency and stability. Various types of nanoparticles have been used for precise treatment, and their thermodynamic-driven reversible self-assembly has led to a broader range of treatment. Currently, several drugs for treating cancers using oligonucleotides have been launched and are available for use.

## General Application of RNAi

Given its target site specificity and therapeutic potential, RNAi is currently used in many research fields and treatments Since 2018, some RNAi drugs have been approved for sale due to their efficacy and safety (Weng et al., 2019). The following parts will briefly describe some applications of RNAi in biomedicine.

### RNAi in Typical Fields

#### Neurodegenerative Disease

Studies have shown that reducing the expression of mutant genes using RNAi technology is a potentially effective strategy for the treatment of dominant hereditary neurodegenerative diseases, and polyglutamine duplication disorder (Boudreau and Davidson, 2006). In spinocerebellar ataxia type I (SCA1), a dominant hereditary polyglutamine dilated disease, mutated ataxin-1 leads to neuronal dysfunction and cell death in some nuclei of the cerebellum and brain stem through a series of actions. Experiments have revealed that mice lacking the Atxn1 gene showed only mild behavioral abnormalities, suggesting that perhaps RNA-mediated knockout of these two alleles in humans is unlikely to lead to loss of gene function and cause other adverse effects (Xia et al., 2005; Gonzalez-Alegre and Paulson, 2007).

#### Cardiovascular Disease

With regard to cardiovascular disease, the application of RNAi primarily includes the study of the following\: 1) cardiac functional genome, 2) myocardial apoptosis, 3) hypertension, 4) hyperlipidemia, and 5) heart failure (Moguilevsky et al., 2004; Soutschek et al., 2004; Watanabe et al., 2004; Vázquez et al., 2005). As many cardiovascular diseases are closely related to cardiac functional gene mutations, which indicates that analyzing specific heart pathogenic genes is necessary. With the help of RNAi, the coding region (exon) or the promoter region of the target gene is controlled by the same promoter with reverse repetition. The transcribed RNA in the transgenic individual forms dsRNA, producing an RNAi effect and resulting in target gene silencing, so as to analyze the function of the target gene (Hamar et al., 2004; Mingshe et al., 2007).

#### Viral Infection

In a relatively short time, RNAi has been demonstrated to be effective against a variety of viruses in cultured cells, including respiratory syncytial virus, the influenza virus, HIV, and hepatitis C virus (Ge et al., 2004; Bennasser et al., 2007; Betáková and Svančarová, 2013). However, more studies regarding the resistance of viral-related HIV-1 genomic RNA to RNAi-mediated degradation in infected cells need to be conducted (Bennasser et al., 2007). In addition, a novel coronavirus, SARS-CoV-2, which causes COVID-19 spread widely around the world in 2020. Due to the rapid infection rate of SARS-CoV-2 and the poor outcomes of conventional non-drug interventions for controlling the spread of the virus, RNAi was tested against the virus. RNAi has been found to be one of the strategies to inhibit the replication of SARS-CoV-2 (Baldassarre et al., 2020). A survey showed that many critically ill patients with COVID-19 died of organ failure caused by a “cytokine storm”. The “cytokine storm” is caused because of the excessive activation of the immune system in response to external factors. The activation releases many cytokines, such as interleukin-17 (IL-17) and tumor necrosis factor, which attack the normal cells of the body and induce severe inflammation. Therefore, interfering with key cytokines is an effective way to minimize life-threatening tissue damage, especially those in lungs. The core role of IL-17 in inducing chemokines may be a feasible target in this respect. Experiments in IL-17RA gene-deficient mouse models have shown that viral infection reduces the migration of immune cells to the lungs, thus lessening the incidence in animals. The levels of pro-inflammatory cytokines TNF- α, IL-1 β and IL-6 in IL-17RA-deficient mice decreased, which indicates the role of master regulation in this regard. Hence, researchers have studied RNAi drugs that target IL-17 to attenuate cytokine storms and minimize unintentional damage (Uludag et al., 2020). To date, no RNAi drugs based on this research direction are available.

#### Cancer

Tumors are the result of the accumulation of various types of gene mutations and the regulation of gene networks formed by the interaction of these mutated genes. Thus, gene therapies are the fundamental treatment. The expression of target genes (vulnerable nodes) is knocked down by RNAi, locating these nodes which are indispensable to tumor maintenance, with low side effects and low risk, blocking the inherent immunosuppression and triggering immune attacks on tumors. Moreover, one of the advantages of RNAi technology is the rapid development of efficacious and targeted drugs for controlling tumor growth. Therefore, cancer is one of the primary objectives of RNAi-based treatment, for the high relations to gene expression and cell proliferation (Mansoori et al., 2014).

### RNAi-Based Tumor Treatment

Compared with traditional gene therapy, RNAi has the benefits of higher silencing efficiency and stability. Hence, it is widely used in tumor gene therapy research. The application of RNAi in cancer is mainly manifested in the following aspects: 1) inhibition of tumor anti-apoptosis genes, 2) study of tumor signal transduction pathway, 3) inhibition of tumor angiogenesis-related factors, 4) the effect on oncogenes, 5) tumor suppressor genes, and 6) reduction of tumor drug resistance.

#### RNAi in Lung Cancer

RNAi therapies are based on key regulatory molecules involved in cellular pathways such as cell proliferation, migration, and apoptosis, which target and deliver therapeutic genes to lung cancer cells effectively through the use of nanocarriers and known biomarkers for lung cancer (Zhang G. et al., 2018). For example, chitosan-derived carbon, a highly efficient fluorescent nanoparticle, linked to a functionalized siRNA, targets the overexpression of the polo-like kinase 1 (PlK1) gene in tumor cells, which is the basic regulatory factor of mitosis. Studies have shown that these nanoparticles effectively reduce the expression of Plk1 and increase the percentage of cell death caused by apoptosis *in vivo* and *in vitro* (Magalhães et al., 2018). Moreover, nanoparticle-mediated RNAi drugs have become a potential approach to overcome the limitations of conventional chemotherapy because of the selective silencing of oncogenes and multidrug resistance-related genes (Kim et al., 2015). Therefore, the characteristics and functions of nanocarriers and biological knowledge of lung cancer have been used to improve the therapeutic effect of RNAi therapy (Magalhães et al., 2018). By determining how to overcome the barrier of nanoparticle-mediated siRNA transmission to carry out gene therapy for lung cancer and comparing the RNAi-based technology on miRNA and siRNA, the potential application and prospect of microRNA in the diagnosis, prognosis, and treatment of lung cancer has been revealed (Wang et al., 2009; Kim et al., 2015; Naghizadeh et al., 2019).

#### RNAi in Pancreatic Cancer

Pancreatic cancer is an invasive and lethal malignant tumor that is usually asymptomatic in the early stage, leading to delayed diagnosis. Exocrine cell carcinoma in the pancreatic duct is called pancreatic ductal adenocarcinoma (PDAC), accounting for 90% of pancreatic cancer cases. Although a large number of experimental treatment strategies are available for PDAC patients, the 5-year survival rate is still low (only about 3–6%) (Passadouro and Faneca, 2016; Rawat et al., 2019). Additionally, it has low resection rate, poor prognosis, and drug resistance to radiotherapy and/or chemotherapy. The new treatment strategy, target gene knockdown therapy, mediated by RNAi has shown a high treatment potential. Moreover, the resistance of pancreatic cancer cells to radiotherapy and chemotherapy is reduced when these therapies are combined with RNAi (Chang, 2007).

In addition, researchers have conducted the following studies: 1) the common uncontrolled microRNA in PDAC and the possible molecular targets in the signaling pathway of pancreatic cancer, 2) the use of nano-gene-silencing drugs to target drug-resistant patients with pancreatic cancer and duodenal homeobox 1 as a specific and potential RNAi target in pancreatic cancer, and 3) the use of siRNA to silence or inhibit kirsten rat sarcoma viral oncogene (KRAS) and abnormally expressed molecules (such as thrombin, CEACAM6, and EphA2). KRAS is a common mutated oncogene in human cancers, such as pancreatic, colon, and lung cancer. The detection of KRAS gene mutations is an important index for tracking the status of oncogenes, highlighting the development and prognosis of various cancers, and determining the effect of radiotherapy and chemotherapy (Chang, 2007; Wu et al., 2014; Passadouro and Faneca, 2016; Kokkinos et al., 2020). Recently, a miRNA-based therapeutic agent has been developed for pancreatic cancer (Rawat et al., 2019). However, given the high genetic heterogeneity in pancreatic tumors, despite the potential of siRNA therapy, there are obstacles that limit clinical applications, such as poor transport capacity across biological barriers, limited cell uptake, degradation, and rapid clearance of drugs. However, nanotechnology provides a broader platform for future studies to address these challenges (Kokkinos et al., 2020).

#### RNAi in Breast Cancer

Breast cancer is the most common cancer in women, and it is also the leading cause of cancer-related deaths. Different genetic changes and gene expression profiles have distinct effects on the development and progression of breast cancer subtypes, outcome of individual cases, and response to treatment. In this context, the possibility of correcting defective genes and regulating gene expression through gene therapy is becoming a potential treatment strategy for breast cancer (Zuo et al., 2017). Genetic modification of target cells is achieved by transferring genes, gene fragments, or oligonucleotides, including siRNAs and miRNAs, whether *in vivo* or *in vitro* (Bottai et al., 2017).

Some preclinical studies have explored RNAi-based strategies for human epidermal growth factor receptor 2+ (HER2+) breast cancer. Two of them, combining siPLK1 with a peptide fusion protein containing HER2 scFv and using PEG-PLA-based nanoparticles that bind to HER2 scFv for targeted siRNA delivery, have shown successful targeted injection (Ngamcherdtrakul et al., 2016). Moreover, in the context of breast cancer, many researchers pursue the idea of targeted delivery of siRNA to combat and overcome chemoresistance in cancer, using miRNAs as a functional marker to determine cell characteristics and regulate the biological activity of breast cancer cells (Jones and Merkel, 2016; Ma et al., 2018).

#### RNAi in Colorectal Cancer

Colorectal cancer (CRC) is the second most common cancer in the western world and the third most common digestive system tumor in China (Lv W. et al., 2006). Furthermore, the current methods of surgery, radiotherapy, and chemotherapy cannot improve the 5-year survival rate by more than 50%. Considering the fact that the neoplastic transformation of colonic epithelial cells is the result of genetic and epigenetic changes, RNAi has been proposed as a new therapeutic strategy. Compared with the conventional therapy, RNAi therapy has the advantages of relatively higher specificity and efficacy as well as lower toxicity and transport difficulties. However, only a few RNAi-based therapies have had clinical trials because of challenges such as transfection, low specificity, low immune response, and unnecessary gene insertion. *In vitro* and *in vivo* trials are still underway to help identify new molecular targets for the application of RNAi in tumor size reduction (Prados et al., 2013).

In addition, the side effects of chemotherapy are devastating and lead to a low quality of life. Therefore, new intracellular targeting methods, such as siRNA and new nano-delivery systems, are expected to achieve high anticancer potential and low adverse reactions because of their high specificity to molecular targets and delivery strategies. Moreover, colon cancer mediates tumorigenesis through several molecular pathways, such as the overexpression of epidermal growth factor receptor (EGFR) family members. When binding to specific ligands, dimerized EGFR transmits mitotic signals to tumor cells, inducing cell proliferation and resistance to apoptosis. Therefore, the knockout of EGFR by siRNA has been considered a potential strategy for the treatment of colon cancer (Binkhathlan and Alshamsan, 2012). The study also found that high expression of PANDAR may represent a new prognostic marker for patients with CRC. Based on previous studies, the overexpression of PANDAR in CRC, breast cancer, and other tissues is related to the decrease in oxygen saturation, indicating its potential as a biomarker of poor prognosis (Rivandi et al., 2019).

#### RNAi in Other Cancers

In addition to the above cancers, RNAi is also widely used in other types of cancers, such as ovarian cancer, hepatocellular cancer (HCC), gastric cancer, and cervical cancer (Jung et al., 2015; Aghamiri et al., 2019; Hajiasgharzadeh et al., 2019; Kieckhaefer et al., 2019; Tam et al., 2019; Malla et al., 2020; Mroweh et al., 2020).

Ovarian cancer is one of the most difficult gynaecological malignant tumors, which has non-specific toxicity and can cause serious side effects. Unfortunately, chemotherapy cannot cure advanced ovarian cancer that degenerates into multidrug-resistant (MDR) ovarian cancer. Currently, co-delivery of anticancer drugs based on nanoparticles and siRNAs targeting different mechanisms of MDR is a cutting-edge strategy in the treatment of ovarian cancer (Aghamiri et al., 2019). Epithelial ovarian cancer (EOC) is confined to the abdominal cavity in most cases, which provides the possibility for intraperitoneal administration of drugs. Therefore, it is possible to study the use of RNAi technology in the treatment of EOC patients because intraperitoneal administration reduces drug isolation in other organs. Moreover, due to the specific silencing of oncogenes and MDR-related genes, nano-siRNA drugs can greatly help to overcome the limitations of chemotherapy (van den Brand et al., 2018; Aghamiri et al., 2019).

In addition, owing to the unique structure of the liver and the availability of some methods of siRNA transport to the liver, this organ has received a lot of attention as a target tissue for siRNA-based treatment (Mroweh et al., 2020). RNAi technology can provide therapeutic benefits for HCC and reduce the development of HCC. The application of RNAi in the treatment of HCC includes RNAi molecules and their vectors, and the success of this intervention mainly depends on the efficiency of the delivery system and the efficiency of RNAi. Therefore, many studies have been carried out on vectors to maximise siRNA delivery and enhance the effectiveness of siRNA-mediated gene silencing in the clinical development of HCC therapy (Hajiasgharzadeh et al., 2019). For example, one study showed that the lipoylation of PEI-based polymers increased the stability of the complex *in vivo* and led to better liver accumulation and knockout in orthotopic HCC xenotransplantation (Zhang Q. Y. et al., 2018). Overall, RNAi technology has an extraordinary prospect in the field of tumor therapy and many such studies are currently underway.

## Challenges in siRNA Delivery

RNAi therapy has shown great potential in treating diseases such as cancer and influenza since its discovery, but siRNAs have not been fully accepted in clinical practice because of their low stability and drug delivery. For example, unmodified and unprotected siRNAs are easily degraded by nuclease and removed by the kidneys. They are also attacked by the immune system (Bartlett and Davis, 2007; Vicentini et al., 2013). In addition, considering the fact that siRNA therapeutics do not work until entering the cytoplasm, many challenges are encountered during delivery.

### Structural Defects in siRNA

Naked siRNAs are unstable in serum and are easily degraded by nucleases. Studies have shown that modification of the ribosome ring and nuclear base improves the stability of siRNAs and reduces the immune and off-target effects (Sipa et al., 2007; Watts et al., 2008; Bramsen et al., 2009). SiRNAs are engulfed by the reticuloendothelial system (RES), which is composed of phagocytes. Polyethylene glycol (PEG) is added to the nanocomposite to reduce its non-specific interaction with phagocytes, thereby reducing phagocytosis (Tokatlian and Segura, 2010). Moreover, considering the fact that the size of siRNAs is smaller than the renal filtration threshold (5–6 nm), siRNAs are rapidly filtered by the kidney and can be fused with nanoparticles to prolong their half-life *in vivo* (Wang K. et al., 2016).

### Vascular Barrier

The siRNA delivery systems penetrate the vascular endothelium to deliver siRNA therapeutic agents to target cells. The tumor capillaries are discontinuous and have large openings, which are conducive for the transmembrane transport of siRNAs in tumor tissues (Wang et al., 2010). The passive accumulation of nanoparticles in cancer tissues, known as the enhanced permeability and retention (EPR) effect, is associated with many factors, including the permeability of tumor blood vessels and half-life of siRNA drugs (Wang J. et al., 2016), occurs in tumor blood vessels (Fang et al., 2011). Additionally, attention should be paid to the size of the carrier and charge on its surface when selecting the carrier of siRNAs as an oversized carrier with excessive net charge will be easily engulfed by phagocytes (Cho et al., 2008).

### Cell Barrier

The cell membrane is a negatively charged phospholipid bilayer consisting of functional proteins. Because naked siRNA is also negatively charged and water soluble, it cannot passively into cells (Wittrup and Lieberman, 2015). However, this limitation is overcome by transmembrane transport by endocytosis (Meade and Dowdy, 2007). Endocytosis primarily involves pinocytosis and phagocytosis. The former is characterized by the fact that the endocytic body encounters the surface of the cell membrane, which then depresses the body and engulfs it into the cytoplasm. In the latter, the membrane extends outwards forming a pseudopod, which swallows the object into the vesicle and fuses with the lysosome. However, endocytosis is targeted. For example, ligand is used as receptor-mediated endocytosis of foliate (Wang et al., 2017). Chemical modification of lipids and cell-penetrating peptides can also be used to enhance cellular uptake.

**FIGURE 2:**
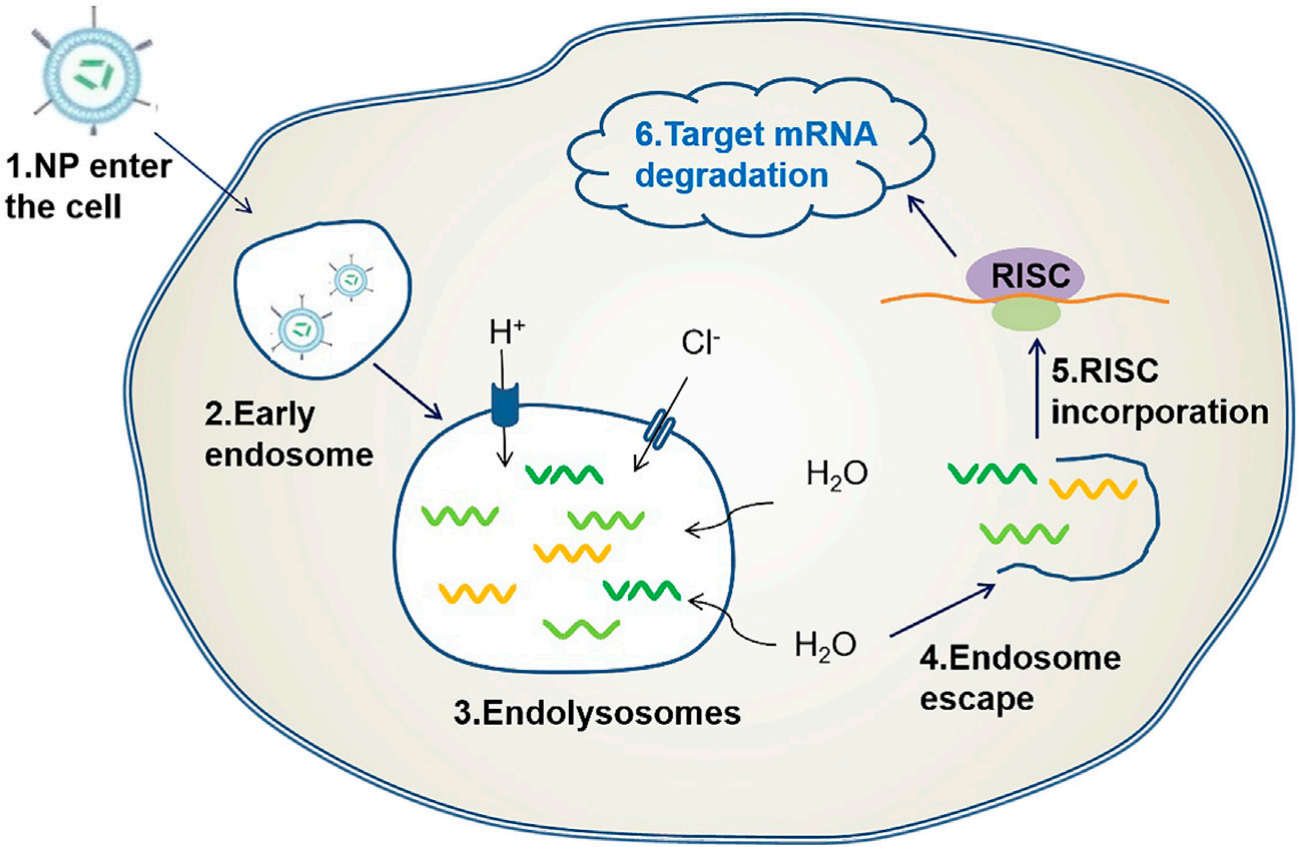
Endosomal escape based on proton sponge effect: When a delivery carrier loaded with siRNA enters the cell, an endosome is formed, followed by an endocytosome. Protons are pumped into the endosome and chloride ions passively enter the endosome causing osmotic pressure to rise, this causes the endosome to break and release the siRNA therapeutic agent.

Another key challenge is the escape from the endosome. Once the delivery system with the siRNA therapeutic agent reaches the target cell, it is encapsulated in the early endosome for transport and fusion with the late endosome. The late endosome causes the pH to become acidic (approximately 5.5) because of the ATPase proton pump. Finally, the late endosome fuses with the lysosome and transports the contents to the lysosome. Therefore, given the low pH (approximately 4.5–5) and the presence of various nucleases, the RNA molecules in the delivery system are degraded. For siRNA therapeutic agents to enter the cytoplasm and exert their effect, they must escape from the endosome. One method of escaping is the proton sponge effect (Figure 2) (Hu et al., 2020). When the above-mentioned effects occur, protons are continuously pumped into the endosome to reduce the pH value, whereas chlorine ions passively enter the endosome and increase its internal osmotic pressure. This causes the endosome to absorb water, burst, and release siRNA therapeutic agents (Sonawane et al., 2003). Another approach is to use ionizable lipids with neutral charges, which cause lysosomes to break apart to release siRNA (Semple et al., 2010).

### Innate Immune Stimulation and Off-Target Effects

RNAi causes off-target effects, which are divided into three categories (Ozpolat et al., 2014): 1) siRNA regulates other transcripts by complementing part of the 3′-UTR sequence (Bramsen et al., 2010), 2) exogenous siRNA competes with endogenous miRNAs for RISC, leading to the saturation of the RNAi mechanism, 3) some synthetic siRNA and its vectors trigger toll-like receptors (TLR3, TLR7, and TLR8) and molecular cascades that cause immune cells to secrete cytokines (Marques and Williams, 2005). Innate immune stimulation may be due to the carrier of siRNA or the RNAi process (Saw and Song, 2020). Although studies have shown that immune stimulation has a positive effect on the treatment of cancer and viral infections, most of them are considered detrimental (Meng and Lu, 2017). Therefore, overcoming this challenge is critical. This could be achieved by using a carrier with low immune stimulation as the delivery system or by modifying siRNA to reduce immune activation such as adding 2′-modified nucleotides to siRNA (Morrissey et al., 2005).

## Delivery Systems

Great efforts have been made to design and develop various carriers to overcome the obstacles in the delivery process. In general, these carriers can be divided into two categories: viral vectors and non-viral vectors. The viral vectors are limited in clinical applications because of their disadvantages, such as high cost and immune stimulation (Bennasser et al., 2007; Mahmoodi Chalbatani et al., 2019). Nanoparticles, a type of non-viral vector, which are 10–1,000 nm in diameter, have attracted considerable attention in recent years. They have many advantages over other delivery systems, such as lower toxicity, better biocompatibility, higher transfection efficiency, lower cost, and large-scale production (Xin et al., 2017). Nanocarriers are divided into organic and inorganic nanoparticles according to their composition. Organic nanoparticles include lipid nanoparticles (LNP) and polymer-based nanoparticles.

### Lipid-Based Delivery System

The composition of lipids, ratio of drugs to lipids, and manufacturing process is optimized to enhance the functionality of LNP. Various lipid-based siRNA delivery systems have been reported, including liposomes, solid LNP, micelles, and emulsions (Whitehead et al., 2009). In fact, LNP is considered a popular vector because of its good stability and high transfection efficiency.

#### Liposomes

**FIGURE 3:**
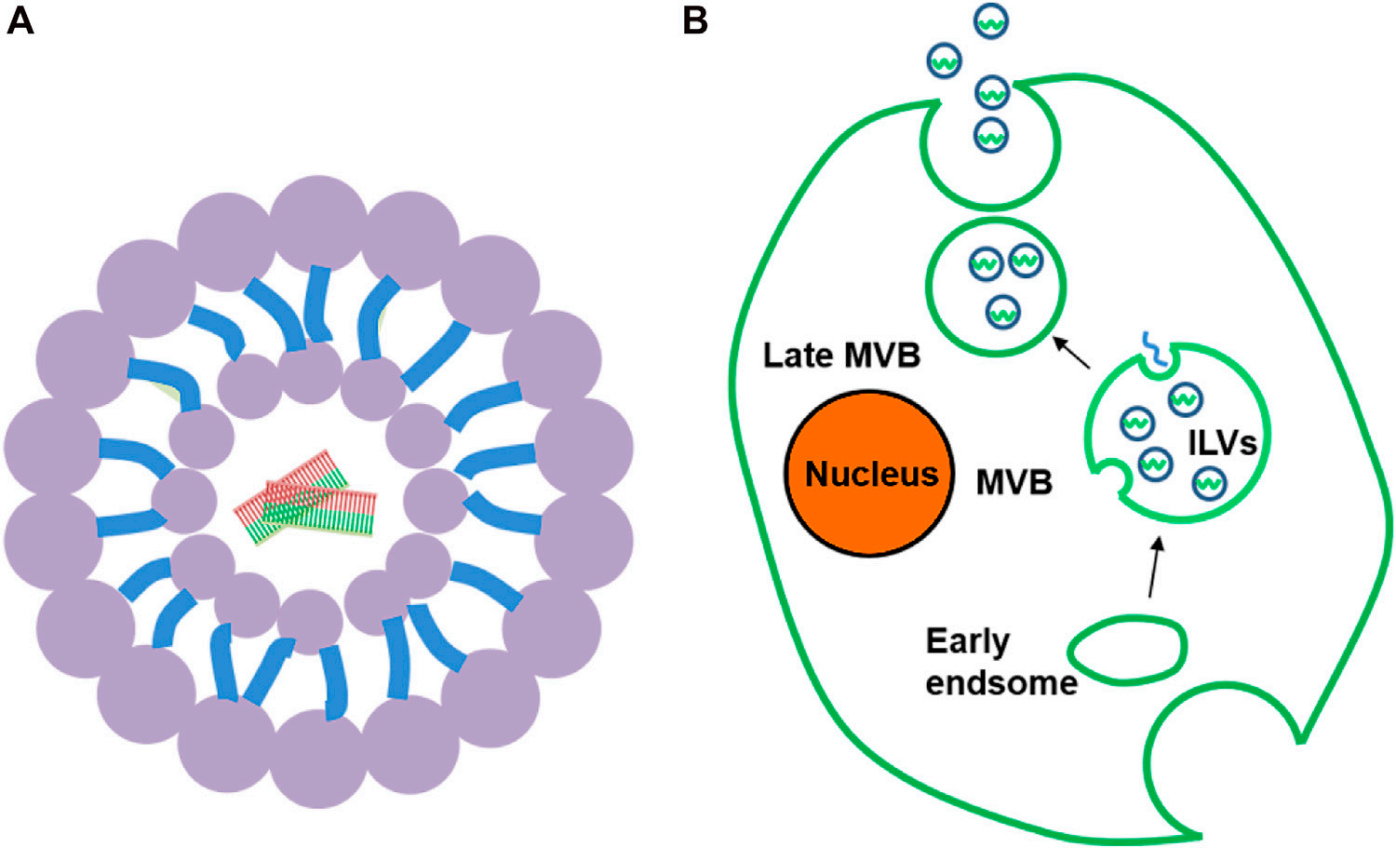
**(A)** Liposome. **(B)**. Formation and secretion of exosomes: In the process of body transition from early to late period, exosomes first bud from plasma membrane to form ILV and further form MVB. MVB then fuses with the plasma membrane to release exosomes.

Liposomes, which are small spherical vesicles composed of a bilayer lipid membrane with an aqueous core inside (Figure 3A), protect drugs from degradation and have the advantages of low toxicity and non-immunogenicity. SCFV-modified LPH (liposomal-polycationic-hyaluronic acid) nanoparticles can be used as carriers of siRNA and miRNA to effectively reduce the expression of target genes in lung metastasis (Chen et al., 2010). In addition, because of the characteristics of hydrophilic and lipophilic drugs, liposomes promote the transfer of small and large molecules and are fused with cell membranes (Pattni et al., 2015). The contents are then released into the cytoplasm. Nucleic acid can form a lipid complex with liposomes through electrostatic interaction, which improves its transfection efficiency and stability (Kim et al., 2006). At present, liposomes used as carriers are classified as positive, negative, and neutral. Cationic liposomes are the most popular because of their high encapsulation efficiency (Ozpolat et al., 2014; Young et al., 2016). Moreover, cationic lipids can prolong the half-life of siRNA in the blood and reduce degradation. However, cationic liposomes may cause systemic toxicity because of the positive charge (Lv H. et al., 2006). The surface of cationic liposomes can be modified with PEG to reduce the positive charge, thereby reducing the immune response and prolonging the circulating half-life. Furthermore, PEG modification induces tumor cell uptake of lipid complexes and loss of endoplasmic reticulum escape, leading to siRNA degradation (Campbell et al., 2002). Neutral liposomes may also be used in place of cationic liposomes. 1,2-Dioleacyl-SN-glycero-3-phosphatidylcholine significantly reduces toxicity and effectively delivers siRNA to target cells (Gewirtz, 2007).

In addition, nucleic acids can form lipid complexes with liposomes through electrostatic interactions, which improve their transfection efficiency and stability (Kim et al., 2006). Stable nucleic acid-lipid particles that consist of a lipid bilayer based on a mixture of fused lipids and cationic lipids containing siRNA are a vital platform for siRNA delivery (Mahmoodi Chalbatani et al., 2019). The lipid bilayer not only promotes cell uptake but also improves intracellular escape ability. Moreover, its surface is also coated with PEG to improve its stability (Rossi, 2006).

#### Solid Lipid Nanoparticles

Given the disadvantages of liposomes, such as easy clearance by RES and instability, studying a new carrier to replace liposomes has been the focus of research since 1990 (Üner and Yener, 2007). Solid LNPs have attracted considerable attention because of their good stability, safety, and easy mass production (Naseri et al., 2015). The average size of SLN is 40–1,000 nm, and it is made up of an internal solid lipid core and an external surfactant (Pardeike et al., 2009; Chauhan et al., 2016). Lipids are solid at the human body and ambient temperature, and can be prepared with steroids, diglycerides, and waxes (Lima et al., 2013). Different combinations of lipids and surface-active substances may affect particle size, drug load, and other properties. SLN has three drug incorporation models: solid solution model, core shell model (rich drug core), and core shell model (rich drug shell) (Pardeshi et al., 2012). SLNs are reported to be markedly successful in food, cosmetics, and gene transfer. In addition, SLNs are administered in a variety of ways, including parenteral administration, oral administration, rectal administration, nasal administration, and respiratory delivery (Uner and Yener, 2007; Pardeshi et al., 2012).

#### Micelle

Micelles are spherical structures composed of monolayer phospholipids with a hydrophobic core and hydrophilic shell, which are automatically assembled by amphiphilic copolymers in water. They are approximately 10–100 nm in diameter, and they can be used as carriers of siRNA and miRNA for cancer treatment (Costa and Torchilin, 2018). The hydrophilic shell reduces the phagocytosis of the nanoparticles by RES and prolongs the half-life of the drug (Yousefpour Marzbali and Yari Khosroushahi, 2017). Amphiphilic copolymers, which are composed of hydrophobic and hydrophilic blocks, are characterized by good stability and biocompatibility (Tiwari et al., 2012). The size of the micelles can be changed by adjusting the hydrophobic blocks of the amphiphilic copolymers, which also helps the nanoparticles to avoid renal filtration (Torchilin, 2006). Studies have shown that micelles have the advantages of low toxicity, prolonged half-life, accurate targeting, and streamlined preparation (Trivedi and Kompella, 2010).

### Polymer-Based Delivery System

Great progress has been made in the research of polymer-based delivery systems as a non-viral vector. Positively charged cationic polymers can form complexes with negatively charged siRNA by electrostatic attraction thus, the applications of polymer-based delivery systems are relatively extensive (de Ilarduya et al., 2010). Many types of cationic polymers have been studied as carriers; one of the types is synthetic polymer (polycation containing cyclodextrin (CD), PEI, etc.) and the other is natural polymer (chitosan) (Xin et al., 2017).

#### Polyvinyl Imine

PEI is one of the most studied cationic polymers for siRNA and miRNA delivery, with straight and branched forms (Singh et al., 2018). PEI has a high cation charge density, which is considered as one of its advantages (Akhtar and Benter, 2007). In the case of Ebola virus infection in guinea pigs, PEI has been shown to have an effective antiviral effect (Akhtar and Benter, 2007). In the mouse model, PEI inhibits HER2 and plays an anti-tumour role (Urban-Klein et al., 2005). However, the cytotoxic effects of unmodified PEI limit its clinical application (Pandey and Sawant, 2016). PEI can be combined with other polymers such as PEG, chitosan, and hyaluronic acid to reduce cytotoxicity (Kim et al., 2008; Kim et al., 2013).

#### Chitosan

Chitosan is a natural polysaccharide with low cytotoxicity, low immunogenicity, and biodegradation. It is electrically positive under weakly acidic conditions and can form complexes with siRNA. Because of the transfection ability of siRNA, chitosan must be modified to improve its transfection efficiency. For example, chitosan modified with polypeptides showed better transfection efficiency (Sun et al., 2017). The ethylene glycol chitosan-PEI-siRNA developed by Huh et al. can accumulate in large quantities in tumors (Huh et al., 2010).

#### Cyclodextrin

CD is a naturally occurring oligosaccharide, which is circular and linked by an α-1,4 glycoside bond. A common derivative of CD is β-CD, and it is characterized by a hydrophilic outer surface and hydrophobic inner chamber, which facilitate the delivery of hydrophobic drugs (Singha et al., 2011). The siRNA is bound to siRNA tightly by electrostatic interaction (Malhotra et al., 2018). Studies have shown that CD modified by the imidazole group contributes to the release and intracellular transfer of siRNA (Kulkarni et al., 2005). CALAA-01 is a targeted nanoparticle system consisting of a CD polymer, human transferrin, and PEG, which inhibits tumor growth by inhibiting the expression of the M2 subunit of ribonucleotide reductase R2 (Davis et al., 2010).

#### Dendritic Polymer

Dendrimers are highly branched artificial macromolecules with a high degree of symmetry, which are mainly composed of the nucleus at the center, multiple branches, and terminal groups (Wu et al., 2013). The end group can be functionalized; providing a variety of functions. On the surface of the polymer, significantly positive amine functional groups are also found, which are conducive for tight binding with siRNA (Liu et al., 2016). Recently, cationic dendrimers have been studied as carriers for siRNA delivery. Based on previous reports, PAMAM and siRNA inhibit TWIST1 expression and silence the target genes (Finlay et al., 2015). Dendrimers can also be used as carriers for therapeutic miRNAs. For example, the expression of EGFR protein in U251 glioma cells was reduced by 90% using PAMAM as the vector of miR-7 (Liu et al., 2013).

#### Exosomes

Exosomes are naturally occurring nanovesicles, which have attracted much attention because of their low immunogenicity, better delivery, higher circulatory stability, and excellent targeting ability (Andaloussi et al., 2013; Alvarez-Erviti et al., 2011). More impressively, it delivers drugs into the cytoplasm by fusing directly with the cell membrane, thereby avoiding the endoplasmic reticulum escape in the endocytic pathway and providing a new way for transmitting siRNA/miRNA (van den Boorn et al., 2011; Lu et al., 2018). Exosomes are spherical, 40–100 nm in diameter, and are composed of lipid bilayer with a variety of lipids and proteins, as well as nucleic acids (Lu et al., 2018). This ideal particle size helps to avoid renal filtration effect and immune system clearance, thus effectively prolonging its half-life (Ren et al., 2016). Almost all cell types can secrete exosomes and they naturally occur in body fluids, including blood, urine, breast milk, and cerebrospinal fluid (Kalani et al., 2014). Generally, the formation of exosomes can be divided into three steps: 1) intracytoplasmic membrane invagination to form vesicles; 2) contains multiple intraluminal vesicles (ILVs) to form multivesicular bodies (MVB); 3) MVB is fused with the plasma membrane to release ILV, which is called an exosome (Théry et al., 2002) (Figure 3B). When exosomes target receptor cells, drugs can be delivered to target cells through cell fusion and endocytosis (Li et al., 2016).

Exosomes have diverse biological functions. For example, they can participate in the immune response and promote antigen presentation (Aharon and Brenner, 2009). Platelet-secreted exosomes may participate in the inflammatory response (Heijnen et al., 1999). Moreover, exosomes can accelerate the development of neurodegenerative diseases by transporting proteins that are incorrectly expressed to other healthy cells (Ghidoni et al., 2008). In some tumor studies, exosomes promote angiogenesis and tumor cell migration (Kucharzewska et al., 2013). To date, many studies have been carried out using exosomes as siRNA/miRNA transporters for the treatment of diseases. MiRNAs transported by exosomes can significantly inhibit the growth of liver tumors (Fonsato et al., 2012). The blood-brain barrier prevents many harmful substances such as bacteria and viruses from entering the brain, but it also prevents many drugs from entering the brain to treat diseases. In a study of zebrafish glioblastoma, exosomes effectively crossed the blood-brain barrier and prevented the expression level of the target gene (Yang et al., 2017). Exosomes also have high potential in the treatment of tumors. Zhang et al. conducted an exosome loaded siRNA experiment to target TGF- 1 and inhibit tumor growth in mice, and found that it could effectively inhibit the growth of tumor tissue (Zhang et al., 2014).

## Launched and Ongoing Drugs

As science and technology develop, new challenges continue to emerge. For instance, although researchers have encountered many setbacks and difficulties in the studies of RNAi, they persisted till they reached their expectations. Many RNAi drugs are entering the market after clinical trials and regulatory approval. Some RNAi drugs that have been launched and tumour-related RNAi drugs that are undergoing preclinical research will be discussed in the next section.

### RNAi Drugs in Cancer

Although there has been remarkable progress regarding the use of RNAi drugs in treating tumors there are still many challenges associated with their use. The only RNAi drug on the market is called rintatolimod, with the United States trade name Ampligen. Rintatolimod is a double-stranded RNA drug first launched (Table 1) in Argentina in 2017 for the treatment of severe myalgic encephalomyelitis/chronic fatigue syndrome. It is not licensed for cancer treatment. However, some progress has been made in preclinical studies. Phase I and I/II clinical trials are ongoing at AIM ImmunoTech (formerly HemispheRx) to evaluate its vaccine adjuvant potential for the treatment of stage II–IV HER2+ breast cancer, triple-negative breast cancer, and several other solid tumors, such as renal cell carcinoma, pancreatic cancer, and ovarian cancer. The molecular mechanism is Toll-Like Receptor 3 (TLR3, Table 1) agonists, and it was found that the activation of TLR3 *in vitro* can induce apoptosis in lung cancer cell lines. In addition, some studies support the use of TLR3 agonists in patients with non-small cell lung cancer (NSCLC) to reactivate the local innate immune response (Bianchi et al., 2020).

**TABLE 1:** Tumour-related RNAi drugs.

Drug name	Clinical advance	Mechanism of action	Therapeutic group
Ampligen (Jiang et al., 2008)	Launched—2017	TLR3 Agonists (Jiang et al., 2008; Iribarren et al., 2016)	Bladder cancer, breast cancer, CRC, ovarian cancer, pancreatic cancer, prostate cancer, renal cancer
Genasense (Han et al., 2019)	Pre-registered	Apoptosis inducers, BCL2	Breast cancer, CRC, gastric cancer, liver cancer, lung cancer, pancreatic cancer, prostate cancer, renal cancer
Lefitolimod	Phase III	TLR9 agonists	CRC, small cell lung cancer
IMO-2125 (Wang et al., 2018)	Phase III	Cytokine, TLR9 agonists	CRC, head and neck cancer, NSCLC
LY-900003 (Villalona-Calero et al., 2004)	Phase III	PKCA	Breast cancer, NSCLC, ovarian cancer
Imetelstat sodium	Phase II/III	Telomerase reverse transcriptase inhibitors	Breast cancer, liver cancer, neurologic cancer, NSCLC, ovarian cancer
Oncomyc-NG	Phase II/III	MYC	Bladder cancer, breast cancer, lung cancer, pancreatic cancer, prostate cancer, renal cancer
BNT-122	Phase II		Bladder cancer, breast cancer, CRC, NSCLC, pancreatic cancer, renal cancer
NCI-4650	Phase II		Digestive/Gastrointestinal cancer
siG12D LODER	Phase II	KRAS (Gly12Asp mutant)	Pancreatic cancer
Danvatirsen	Phase II	STAT3	Bladder cancer, CRC, liver cancer, NSCLC, pancreatic cancer
ATU-027	Phase II	PKN3	Digestive/Gastrointestinal cancer, pancreatic cancer
EGEN-001	Phase II		Brain cancer, CRC, ovarian cancer, pancreatic cancer
Apatorsen sodium	Phase II	Heat shock protein 27, HSPB1	Bladder cancer, breast cancer NSCLC, ovarian cancer, pancreatic cancer, prostate cancer
ISIS-EIF4ERx	Phase II	EIF4E	NSCLC, prostate cancer
AEG-35156	Phase II	BIRC4	Breast cancer, liver cancer, NSCLC, pancreatic cancer
ACT-GRO-777	Phase II	Anti-nucleolin (NCL)	Lung cancer, pancreatic cancer, renal cancer
dSLIM	Phase II	TLR9 agonists	CRC, renal cancer
ISIS-23722	Phase II	BIRC5 (survivin)	NSCLC, prostate cancer
GTI-2040	Phase II	RRM2	Bladder cancer, breast cancer CRC, NSCLC, prostate cancer, renal cancer
Agatolimod sodium	Phase II	TLR9 agonists	Breast cancer, NSCLC, prostate cancer, renal cancer
ISIS-2503	Phase II	HRAS	Breast cancer, CRC, NSCLC, pancreatic cancer
CGP-69846A	Phase II	RAF1	Breast cancer, ovarian cancer
Poly I: CLC	Phase II	TLR3 agonists	CRC, liver cancer, neurologic cancer, ovarian cancer, pancreatic cancer, prostate cancer
ARB-1598	Phase I	TLR9 agonists	CRC, head and neck cancer, NSCLC
Emapticap pegol	Phase I/II	Anti-CCL2 (C-C motif chemokine 2; MCP-1)	Pancreatic cancer, solid tumors
Archexin	Discontinued	AKT1	Liver cancer, ovarian cancer, pancreatic cancer, renal cancer
AS TRPM2 ODN	Discontinued	CLU	Breast cancer, NSCLC, prostate cancer

Genasense (oblimersen) is an antisense oligonucleotide drug specific to Bcl-2 (Table 1). Bcl-2 protein inhibits apoptosis and is upregulated in many cancers (Gjertsen et al., 2007). Bcl-2 is the first mammalian apoptosis regulator (Han et al., 2019). Genasense can specifically bind to human Bcl-2 mRNA, which leads to the catalytic degradation of Bcl-2 mRNA and reduces the translation of Bcl-2 protein. Therefore, researchers can use mRNA degradation strategies and small inhibitory molecules for targeted Bcl-2 therapy in a variety of cancers, such as breast cancer, colon cancer, and prostate cancer (Li et al., 2016). Moreover, preclinical studies have shown that the combination of Bcl-2 antisense and chemotherapy improved the anti-tumour response, thereby increasing the apoptosis of tumor cells and improving the survival rate. In brief, although cancer-related RNAi drugs have not been marketed, they still have tremendous therapeutic potential. Finally, the summary of the tumour-related RNAi drugs is shown in Table 1.

### RNAi Drugs in Other Diseases

Since the first RNAi drug was launched, some RNAi drugs (such as HBV-ISS, mipomersen sodium, nusinesen, inotersen, volanesorsen, patisiran/onpattro, pegaptanib sodium, and viltolarsen) have been used in clinical treatment and have shown unparalleled advantages over other types of drugs (Moshfeghi and Puliafito, 2005; Crooke et al., 2018; Hoy, 2018; Keam, 2018; Mathew and Wang, 2019; Parham and Goldberg, 2019; Weng et al., 2019; Dhillon, 2020; Esan and Wierzbicki, 2020). Among these, nusinesen will be used as an example to illustrate theefficacy of RNAi drugs in clinical applications.

Nusinesen is used to treat spinal muscular atrophy (SMA), an autosomal recessive neuromuscular disease caused by homozygous loss or mutation of the survival motor neuron 1 (SMN1) gene (Chiriboga, 2017; Crooke et al., 2018; Li, 2020). Before the advent of nusinesen, SMA was a common genetic cause of infant death because effective treatment was not available for this serious disease. Following the development of nusinesen, the symptoms of patients have improved. For example, 92% of infants treated with nusinesen were able to sit without support before the onset of symptoms, a milestone that type1 SMA infants never reached before the introduction of nusinesen treatment. In addition, 50% of patients treated with nusinesen were able to walk without support (Crooke et al., 2018). However, nusinesen also has potential disadvantages, such as the high cost of drugs and potential risks of repeated lumbar punctures. For most patients, the benefits outweigh the risks (Chiriboga, 2017).

## Outlook

To date, cancer remains the leading cause of death in humans, Further uncontrolled proliferation and aggressiveness of cancer cells render many medical treatments ineffective. The remarkable gene silencing capabilities of siRNA therapy offer a glimpse into the future, offering new strategies for the treatment of cancer and other diseases. After decades of development, siRNA therapy has made excellent improvements regarding targeting ability and delivery efficiency. Many pharmaceutical companies and research institutes have also developed a variety of RNAi drugs, but only a few have passed phase I clinical trials. Challenges such as stability, stimulation of innate immune stimuli, off-target effects, and safety concerns continue to prevent siRNA-based drugs from reaching their full potential. A large number of studies are expected to address these challenges. It is believed that RNAi therapy, which exerts its effect *via* gene silencing, will aid in faster and better treatment of diseases such as cancer, and bring hope to patients, physicians, and researchers.

## Glossary

AuNPs, gold nanoparticles: CNTs,
CaP: calcium phosphate nanoparticles
CD: cyclodextrin
CNTs: carbon nanotubes
CRC: colorectal cancer
DOPC: 1,2-dioleacyl-sn-glycero-3-phosphatidylcholine
dsRNA: double-stranded RNA
EPR: electron paramagnetic resonance
GBM: glioblastoma
HCV: hepatitis c virus
lncRNA: long non-coding RNA
LNP: lipid nanoparticles
ME/CFS: myalgic encephalomyelitis/chronic fatigue syndrome
miRNA: microRNA
MNPs: magnetic nanoparticles
MSNs: mesoporous silica nanoparticles
ncRNA: non-coding RNA
NSCLC: non-small cell lung cancer
OS: overall survival
PDAC: pancreatic ductal adenocarcinoma
PEG: polyethylene glycol
PEI: polyvinyl imine
QD: quantum dots
RES: reticuloendothelial system
RISC: RNA-induced silencing complex
RNAi: RNA interference
RSV: respiratory syncytial virus
siRNA: small interference RNA
SLNs: solid lipid nanoparticles
SMN1: survival motor neuron 1
SMA: spinal muscular atrophy
SNALP: stable nucleic acid-lipid particles
